# Ethnobotanical study of traditional medicinal plants used by the local Gamo people in Boreda Abaya District, Gamo Zone, southern Ethiopia

**DOI:** 10.1186/s13002-024-00666-z

**Published:** 2024-02-28

**Authors:** Juhar Zemede, Tegenu Mekuria, Clintone Onyango Ochieng, Guy Eric Onjalalaina, Guang-Wan Hu

**Affiliations:** 1 CAS Key Laboratory of Plant Germplasm and Specialty Agriculture, Wuhan Botanical Garden, Chinese Academy of Sciences, Wuhan, 430074 China; 2 Sino-Africa Joint Research Center, Chinese Academy of Sciences, Wuhan, 430074 China; 3 University of Chinese Academy of Sciences, Beijing, 100049 China; 4 Hubei Jiangxia Laboratory, Wuhan, 430200 China

**Keywords:** Boreda Abaya, Gamo people, Indigenous traditional knowledge, Wagas

## Abstract

**Background:**

Medicinal plants have been used for centuries and are still relied upon by over 80% of the Ethiopian population. The people of Gamo, southern Ethiopia, have a rich cultural and traditional lifestyle with a long history of using plant resources for various uses including traditional herbal medicine. However, their traditional knowledge of traditional medicinal plants in Boreda Abaya District has not been explored Ethnobotanically yet, despite preserving diverse indigenous traditional medicinal plants. Hence, the study aimed to document and analyze traditional medicinal plants and associated traditional knowledge and practices used by local people.

**Materials and methods:**

Quantitative ethnobotanical data were collected via semi-structured interviews, face-to-face conversations, group discussions, and guided field trips between September 2022 and February 2023. In total, 92 informants participated, of which 25 were key informants. Quantitative data indices (informant consensus factor—ICF—and use report—Ur) were computed by MS Excel spreadsheet software. Scientific names of medicinal plants were checked via World Flora Online.

**Results:**

In the present study, we recorded 188 traditional medicinal plant species belonging to 163 genera and 73 plant families. Lamiaceae (16 species), Asteraceae (16 species), Fabaceae (11 species), and Euphorbiaceae (8 species) contributed highest number of species and were found to be predominant family in the area. Leaves and seeds were most frequently used plant parts, and pounding (46%) was the main method to prepare remedies. The sudden sickness disease category scored the highest consensus (ICF: 0.35), followed by blood and circulatory-related disease categories (ICF: 0.33). The highest number of plant taxa (61 species) used to treat dermal disease has a 71-use report score, while fewer plant taxa (21 species) were utilized to treat genitourinary system-related disease category, having 25 use reports. *Ocimum lamiifolium* (Ur:56) and *Moringa stenopetala* (Ur:51) are widely used species and received highest use report value.

**Conclusion:**

Gamo people possess extensive traditional knowledge of ethnomedicine. The region's vegetation hosts diverse medicinal species, but deforestation, agriculture, and droughts threaten them. Local conservation practices require scientific support, prioritizing species having higher use reports (Ur), and in-depth investigations of promising species for drug development are essential.

## Background

The human search for drugs goes back to ancient times, and awareness of medicinal plant usage results from many years of struggles against diseases and humans learning to pursue drugs from different parts of plants [1]. The human–plant relationship is not limited to food, clothes, and shelter but extends to health protection [2]. Despite the increasing growth and development of the pharmaceutical industry, the world still consumes ethnomedicine to provide medical care for basic ailments [3]. It is widely reported that about 95% of traditional medicines are sourced from plants and their derivatives [4]. In Ethiopia, traditional plant medicine was used long ago to control various diseases afflicting human and livestock health. Most traditional knowledge is transferred orally, and practitioners are crucial in transferring traditional medicinal knowledge [5]. Some of the traditional practices implemented in Ethiopia include "bone setters" (Wogesha in Amharic), "birth attendants" (Yelimed awalajoch), "tooth extractors," "herbalists," and other spiritual healers such as "Debtera," "Wuqabe," "Kalicha," and "Rukia" (spirit treatments) and major plant-based traditional knowledge has been transferred over generation orally [6, 7].

Traditional medicine has been a significant part of Ethiopia's healthcare system since ancient times [8]. More than 80% of the population relies on traditional medicine for their healthcare needs [9]. These can be attributed to the fact that it is culturally accepted, affordable, cost-effective, and accessible. Additionally, limited access to modern healthcare services in many parts of the country means that rural communities rely on traditional medicine for their primary healthcare [10]. Despite the significant role played by medicinal plants in supporting national primary healthcare, there have been fewer attempts to document and validate the associated knowledge [9]. The existence of interacted culture, ethnolinguistic communities, and geographical diversity blesses the country with the accumulated wisdom of traditional medicines (TMs) which is not well explored, studied, and developed [9]. A limited number (about 1000) of identified medicinal plant species are reported in the Ethiopian Flora; however, many others have not yet been explored and identified [11].

The southern and southwestern parts of the country were enriched with a greater concentration of medicinal plants following the concentration of biological and cultural diversity [12]. However, this rich medicinal plant knowledge is seriously threatened due to deforestation, environmental degradation, and increased population. These serious factors threatened the country's forest, which serves as a source of medicinal plants, causing a loss of indigenous knowledge [13].

Gamo people have a close connection with plants and a traditional lifestyle in the countryside [14]. The vegetation in the region, including 272 sacred groves, contains plenty of medicinal plants [15]. Although different ethnobotanical documentation about several ethnic groups has been published during the past decades in Ethiopia, few ethnobotanical studies have been conducted in Gamo Zone and none in the Boreda Abaya area. It is therefore important to conduct survey to document the medicinal plants and associated indigenous knowledge in Boreda Abaya District. In addition, there is a limitation of infrastructure in the area, including health facilities and schools as compared to other parts of the country; in contrast, the area is rich in diverse and relatively intact traditional cultures and has better forest cover. It is, therefore, crucial to document traditional medicinal plants for local healthcare, and exploring unreached areas can help to update and enrich the flora diversity of the region. Therefore, this study aimed to (i) collect, identify, and document medicinal plants and associated indigenous knowledge of the local people used to treat various human and livestock ailments in the study area, (ii) identify and document candidate medicinal plants used in the study area, and (iii) identify major threatening factor of medicinal plants and recommending feasible conservation methods in the area. The study provides basic information on Ethnomedicine and traditional knowledge of local people in the area, which is useful for primary health care promotion and update of regional flora.

## Materials and methods

### Description of study site

**Fig. 1:**
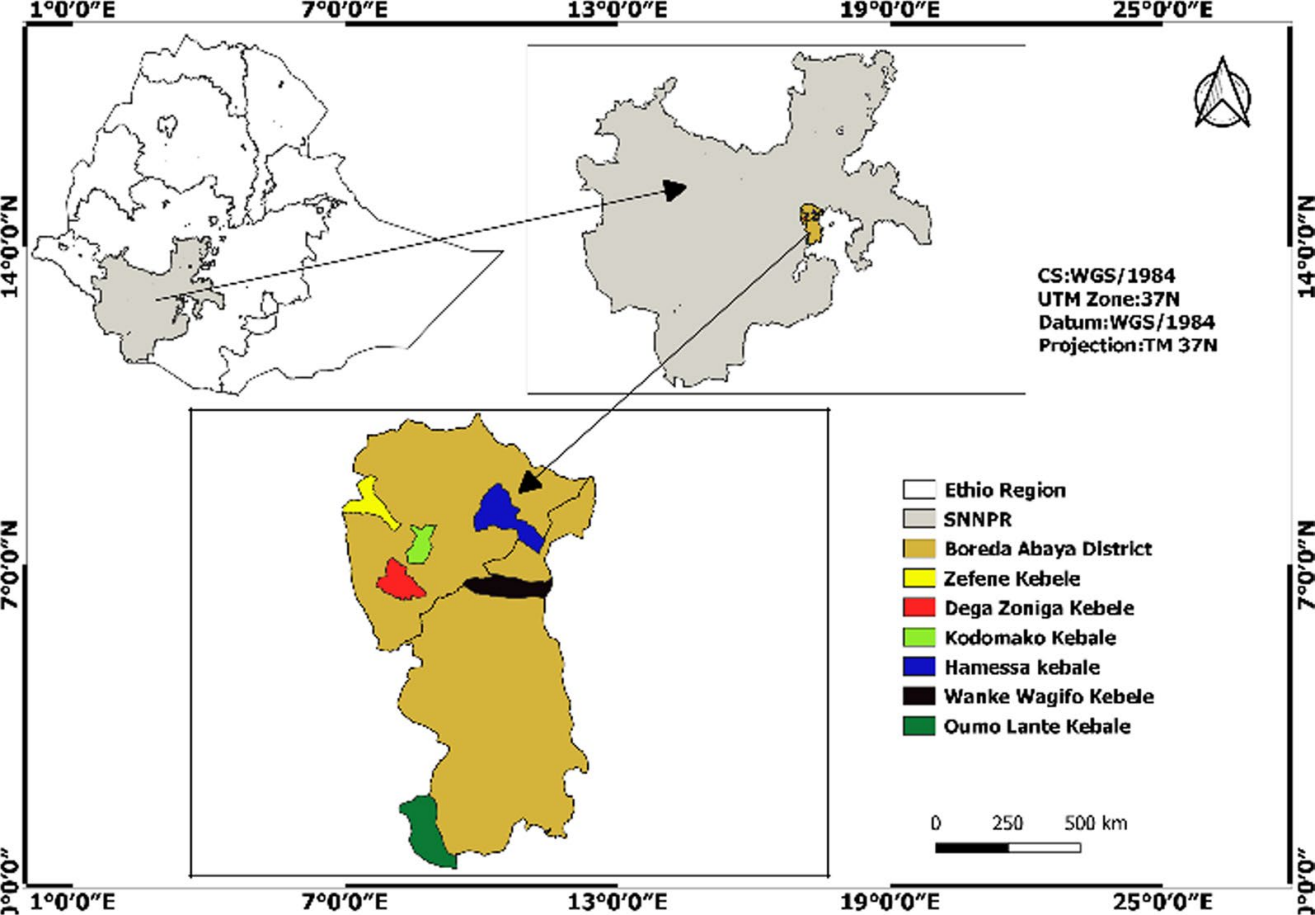
Map of study site

Boreda Abaya is located at 20° 20′ 0′′ N and 37° 15′ 0′′ E in Gamo Zone of southern Ethiopia. It is one of the largest districts in the area but recently disintegrated into two districts, namely Boreda and Mirab Abaya (Fig. 1). It is about 460 km to the southern direction from Addis Ababa (capital city). Gamo people are the dominant indigenous peoples in the area (83.74%), followed by Welayta (10.06%) and Amhara (2.6%), and their language is Gamogna, which belongs to the Omotic language, and majorly follow Christianity religion. They are part of many Omotic groups living in Ethiopia's current southern regional state [16, 17].

Mixed agriculture and weaving are widely practiced among Gamo people. The people cultivate a variety of crops such as teff, maize, sorghum, coffee, yam, cassava, mango, banana enset, sweet potato, and taro [18]. Fruit trees such as bananas, papaya, mango, and avocado are widely cultivated [19]. Spices like turmeric, ginger and cardamom are also produced. For instance, *Artemisia absinthium*, locally called “Arita”, a known medicinal plant in the area is produced on a large area of land for income source. People rear cattle, sheep, goats, and poultry. Skin-related diseases, malaria, intestinal parasitic infections, respiratory tract infections, and typhoid fever are the major public health problems in the district (unpublished data, Boreda Woreda Health Office, 2023). Blackleg, chicken pox, “Gend,”/ shivering are some of the domestic animal diseases (unpublished data, Boreda Woreda Agriculture Office, 2023).

Topographically, Boreda Abaya has three agroecological zones, namely lowland, midland, and highlands, with an elevation between 1100 and 2942 m.a.s.l. It has an estimated area of 1,322.04 square kilometers. The districts' total demography is estimated at 276,249; 139,249 men and 137,000 are females, and only about 4% to 7.78% are urban dwellers. It receives an annual rainfall range between 900 and 2600 mm in bimodal regimes; the first round of rain occurs between March and May, and the second round occurs from June to August. The temperature ranges between 22.5 and 27 °C.

### Data collection

Ethnobotanical data were collected between September 2022 and February 2023 through pre-planned semi-structured questionnaires, face-to-face interviews, field walks, and group discussions [20–22]. With local elders,' herbalists,’ and agricultural DA consultation, six vegetation potential kebeles (minor administrative level in Ethiopia), Kodomoko, Dega zonga, Zefene, Hamesa, Uomo lante, and Wanke-Wajifo were targeted in our field trips and other places randomly selected based on availability of herbal medicine and practitioners (Table 1). In total, 92 residents participated, and 25 of them were key informants. Key informants were interviewed for an extended time to gain detailed knowledge about medicinal plants, and they were supposed to be knowledgeable practitioners [23]. The remaining 52 respondents were chosen randomly by giving a number to each household in order, and one person from each house was interviewed. The data are primarily sourced from key informants since they are expected to be knowledgeable about herbal medicine. During the field trip, we collected information on ethnobotanical data such as local plant names, plant parts used, habitat, preparation methods, modes of application, routes of administration, treated disease type, multipurpose uses, threatening factors, and availability of medicinal plants.

**Table 1:** Specifically visited site within Boreda Abaya District of Gamo Zone, southern Ethiopia

Selected site /kebeles	Agroecology	Ethnicity	Language	GPS coordinates	Altitude range (m.a.s.l)
Degazonga	Dega (Highland)	Gamo	Gamogna, Amharic	62°71′9'' N/37°37′17'' E	2200–2942
Kodomoko	Waine dega (midland)	Gamo	Gamogna, Amharic	62°84′6'' N/37°39′9'' E	1850–2200
Zefene zuria	Weine dega (midland)	Gamo, Wolaita and Amhara	Gamogna, Wolaytegna, Amharic	63°11′6'' N/37°41′52'' E	1500–1850
Hamessa (Xalxalle)	Kola (lowland)	Gamo, Wolaita	Gamogna, Amharic	63°11′1'' N/37°41′41'' E	1100- 1500
Umo lante	Kola (Lowland)	Gamo	Gamogna, Amharic	61°54′3'' N/37°45′57'' E	1100 -1500
Wanke Wajifo	Kola (lowland)	Gamo, Wolaita	Gamogna, Amharic	69°36′0'' N/37°39′48'' E	1100 -1500

### Ethical considerations

Supportive letters were written from Shashemene Botanical Garden to concerned bodies, such as the District Agriculture Office, District administrators, and Kebele administrators, before field trips. We ensured that ethical principles were considered; all herbalists were informed that the purpose of the study was for academic purposes and ethical approval was obtained to ensure confidentiality before conducting interviews. During our research, we maintained the confidentiality of local communities' secrets, knowledge, and taboos while recording notes [22].

### Plant specimen collection/vouchers

During field trips, we collected plant specimens of medicinal plants with the help of herbalists and development agent experts. Two to three specimens from each species were collected to ensure the collection's authenticity. We attached collecting labels with the collection number and collector names. The specimens were correctly placed in the middle of two or three pieces of locally made blotting paper, with some facing up and some facing down to capture both sides. Finally, they are held together and tightened by a specimen presser and holder. The vouchers were dried under sunlight by placing holder side face toward the sun and aerated to check insect strains.

The collected specimens were identified and verified at the herbarium of the Ethiopian Biodiversity Institute/Shashemene Botanical Garden, using taxonomic keys and descriptions from the relevant volumes of the Flora Book of Ethiopia and Eritrea [24–26]. A visual comparison of the specimen with authenticated specimens was conducted to authenticate the identification. The voucher is preserved in the Shashemene Botanical Garden plant herbarium (SBGH). The scientific names, families, and their authors' names of recorded plants were checked in the International Code of Nomenclature for algae, fungi, and plants, World Flora online: https://wfoplantlist.org/, https://powo.science.kew.org/ and Natural database of Africa (NDA). In our field trip, we used a field guidebook of useful trees and shrubs for Ethiopia [24].

### Data analysis

Collected data were analyzed by following the techniques in Martin [27] and Höft et al. [28]. Ethnobotanical data were summarized and analyzed on Microsoft Office Excel using descriptive statistical methods. Quantitative data analysis was conducted using the informant consensus factor (ICF) and use reports (number of citations or mentions). Fidelity level (FL) for some species is computed for additional information. Accordingly, the informant consensus factor was computed following the formula, ICF = Nur-Nt/Nur-1, where ICF = informant consensus factor, Nur = number of species used for each category, and Nt = the number of species used for all ailments. Its values range from 0 to 1, and when values are close to 1, it indicates a high consensus on plant species used against a disease category, and 0 possesses an opposite significance [29]

The mentioned disease conditions were grouped into nine major disease categories based on their sign and symptoms, pathogenic agents, and human or animal parts they attack. The relative therapeutic capacity of medicinal plants used to treat diseases was determined by fidelity level and computed as FL = (*N*/*n*) * 100, where N is the number of informants that claim the use of a species to treat a particular disease and n is the number of informants that use the plant to treat any ailments. A higher FL level indicates high usage of a medical plant for a particular disease, while a low FL level confirms a wide range of medicinal uses but a low frequency for each ailment.

## Results and discussion

### Demographic features of the respondents

The study took place in Boreda Abaya, where most local people belong to the Gamo ethnic group. Of 92 residents who participated in the field trips, males account for a higher proportion than females (76.1%, mentions 136 species) due to the cultural expectation that women primarily work at home while males work in the field (Table 2). The number of females was 22, covering 23.9% and mentions 52 species. The age group of respondents mostly belonged to the 41–60 years old category (41.3%, mentions 108 species), followed by the 20–40 age group (31.5%, mentions 32 species), and the minor age group was 61–84, covering 27.2% and mentions 48 species. This indicates that while older people cite more medicinal plants, their number is less due to aging.

**Table 2:** General background of informants and species reported features

Features	Number of informants	Proportion (%)	Number of species reported
*Gender*			
Male	70	76.1	136
Female	22	23.9	52
*Age*			
20–40	29	31.5	32
41–60	38	41.3	108
61–84	25	27.2	48
*Education level*			
Illiterates	65	70.7	129
Able to read and write	15	16.3	48
Primary school	2	2.2	3
Secondary school	5	5.4	3
College	5	5.4	5
*Occupations*			
Farmers and herbalist	70	76.1	110
Herbalists	9	9.8	67
Employed	6	6.5	5
Students	7	7.6	6

Most informants are illiterate (70.7%, mentions 129 species), with some able to read and write (16.3%, mentions 48 species), attend primary school (2.2%, mentions 3 species), secondary school (5.4%, mentions 3 species), or college (5.4%, mentions 5 species). Farmers and healers account for more occupations (76.1%). A few are only employed in traditional medicine (9.8%), some are students (7.6%), and others are government employees (6.5%).

In the study, it was observed that individuals belonging to the category of farmers and herbalists showed a greater tendency to mention medicinal plants (mentions 110 species) as compared to other informants (Table 2). Informants employed on herbal treatments alone mentioned 67 species. Notably, the highest number of medicinal plants shared by a single healer was 36. Conversely, those who were employed (mentions 5 species) and younger (mentions 6 species) tended to mention 1–2 medicinal species, while farmers and older residents cited a wider range of species. However, it is important to note that it was difficult to obtain comprehensive information regarding traditional medicine from young people because of their limited knowledge in this area. Even though older people mentioned more species, they faced obstacles in accessing those species due to aging and the deforestation of nearby forests. The lack of conventional knowledge among young people causes a risk of the disappearance of traditional knowledge of medicinal plants [30, 31].

### Taxonomy, floral diversity, and life form of Gamo medicinal plants

**Fig. 2:**
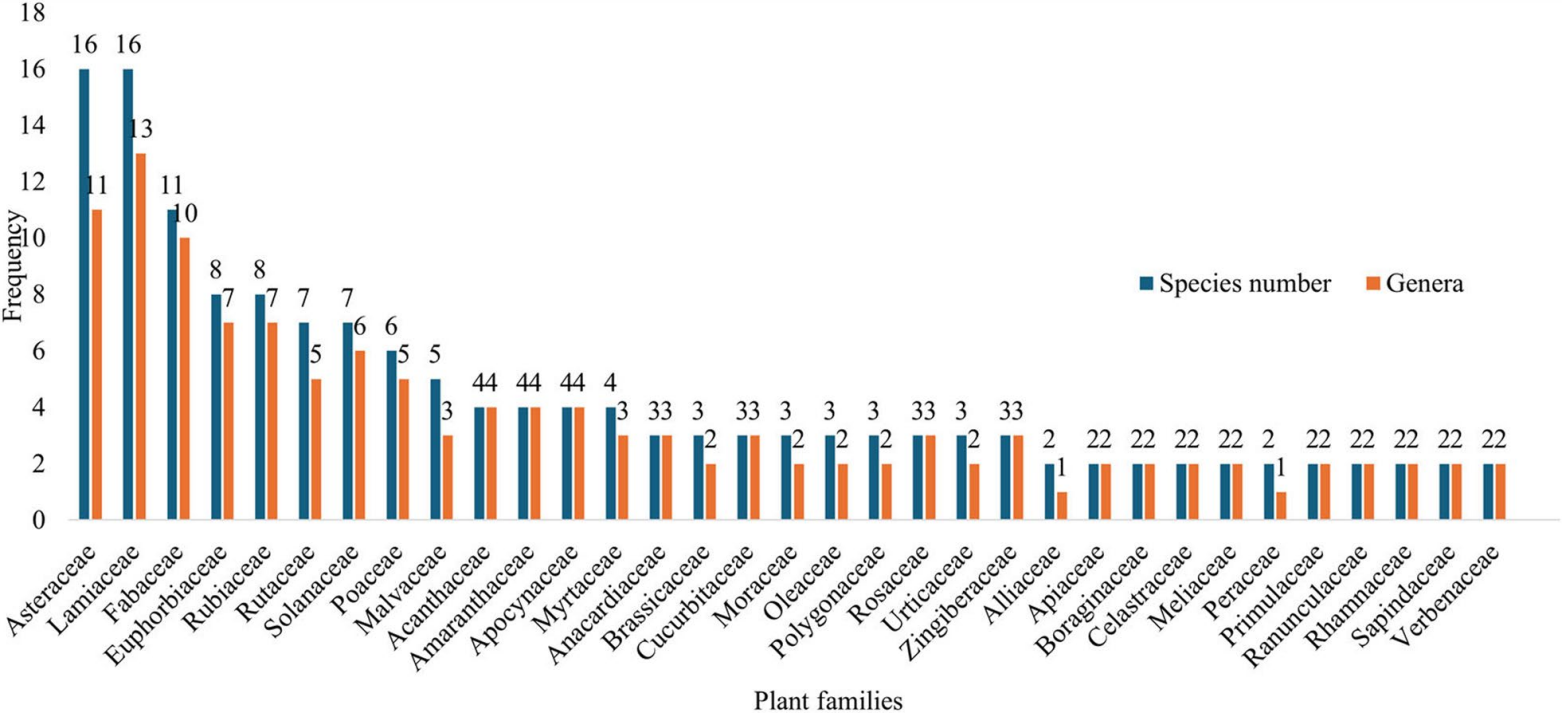
Distribution of predominant medicinal plant families in the Boreda Abaya District

In total, 188 medicinal plants belonging to 163 genera and 73 families were recorded in the present study (Table 3**)**. Many of those species were utilized for human diseases (123 species). Fewer species (11 species) were recorded for domestic animal disease treatments, and 54 species were used for human and livestock diseases. These results show that local healers prioritize human diseases and utilize diverse plant species in treatment, resulting in richer traditional knowledge. Furthermore, the large number of recorded species indicates that the vegetation of the study area is a reservoir for diverse medicinal species, supporting their critical importance in plant-based traditional medicine in fulfilling the needs for primary healthcare. Prominent plant families, Lamiaceae (16 species), Asteraceae (16 species), Euphorbiaceae (11 species), and Fabaceae (11 species), contributed a significant number of species and well-represented families in the area (Fig. 2). Species from those families can survive in various ecology and widely distributed to the local environment [15]. Those families host larger species composition in different ethnobotanical surveys conducted elsewhere in the country [32, 33]. They also accounted for a more significant portion of the country's Flora due to widely dispersed, readily available, and more utilized families [31, 34–38]. Species of those families are widely used due to their essential phytochemical compounds, which provide significant health benefits [39]. For example, *Ocimum* species (Lamiaceae) are rich sources of tannins, phenolic acids, anthocyanins, phytosterols, policosanol, and essential oils, which have potential biological activities such as antimicrobial, antioxidant, anticancer, and anti-inflammatory properties [39].

**Table 3:** Ethnomedicinal plants used by Gamo people of Boreda Abaya District and their traditional methods of remedy preparation (sorted by family name)

Family	Scientific name /species use report (Ur)	Local Name	Treated disease type	PU	Hb/Hbt	MP	Application mode	RA	V.N	Uses
Acanthaceae	*Acanthus sennii* Chiov. (5)	Kosheshlt	Cancer, Malaria	Leaves	S/W	Pounding and filtering, then mix with butter	Drenching	Oral	JZ082	Hm
	*Hypoestes forskaolii* (Vahl) R.Br. (1)	Dergu	Diabetes	Leaves	H/W	Pounding	Drenching	Oral	JZ043	Hm
	*Justicia schimperi* (Hochst.) Dandy (21)	Algi	Tapeworm	Leave, Seed	CL/W	Pounding	Drenching	Oral	JZ006	Hm
	*Thunbergia abyssinica* Turrill (3)	Abeba hareg	Fever, wart	Leaves, fruit	CL/W	Pounding	Drenching	Oral	JZ001	Hm
Alliaceae	*Allium cepa* L. (11)	key tumo	Hypertension	Bulb	H/Hg	Cooking	Eating	Oral	JZ169	Hm
	*Allium sativum* L. (20)	Nech shinkurt	Malaria, common cold, sexual impotence, hypertension	Bulb	H/Hg	Pounding, crushing *Zingiber officinale*	Eating	Oral, Nasal	JZ053	Both
Aloaceae	*Aloe* spps (23)	Ret	Malaria	Latex	H/Rs	Cutting and eating after removing the skin from the bulb	Eating	Oral	JZ168	Hm
Amaranthaceae	*Achyranthes aspera var. sicula* L*.* (1)	Telenji	Headache, fever	Leaves	H/W	Squeezing	Sniffing	Nasal	JZ134	Hm
	*Alternanthera pungens* Kunth (2)	kindicho	Kidney, mate organ burn (male)	Leave	H/W	Cooking	Drenching	Oral	JZ186	Hm
	*Beta vulgaris* L.(4)	Key sir	Hypertension, migraine	Root, leave	H/Hg	Cooking	Eating	Oral	JZ075	Hm
	*Celosia trigyna* L. (5)	Majimala	Internal parasite	Leave	H/W	Pounding	Rubbing	Oral	JZ089	Hm
Anacardiaceae	*Mangifera indica* L. (15)	Mango	Heartburn, diabetes, gastric	Fruit	T/Fl	Cutting and eating fruits directly	Eating	Oral	JZ091	Hm
	*Rhus ruspolii* Engl. (1)	Maldaye	Trachoma/eye disease	Leaves, root	S/W	Grinding and pounding	Rubbing around eye	Optical	JZ090	Lv
	*Sclerocarya birrea* (A. Rich.) Hochst. (1)	Yebereha lomii	Diarrhea	Fruit	T/W	Cutting and eating fruits	Eating	Oral	JZ147	Both
Annonaceae	*Annona senegalensis* Pers. (8)	Gishta	Gastric, heart problems, diabetes, toothache, pneumonia	Fruit, Leave, bark	S/Hg	Cutting the fruit, pounding the bark	Eating	Oral	JZ061	Hm
Apiaceae	*Coriandrum sativum* L. (9)	Debo	Malaria	Seed	H/Hg	Powdering and mix with water	Drenching	Oral	JZ179	Hm
	*Foeniculum vulgare* Mill. (15)	Katikala	kidney stone, headache, asthma	Leave, flower, seed	H/Hg	Dry and pounding	Chewing, drenching	Oral	JZ072	Hm
Apocynaceae	*Acokanthera schimperi* (A.DC.) Schweinf. (11)	Yeshincha	Wound, dermatitis	Leave, Roots	T/W	Crushing, powdering, and cooking	Eating, rubbing	Oral, dermal	JZ151	Both
	*Leptadenia hastata* Vatke (1)	Bosatura	Hypertension	Leave	CL/W	Pounding and mixing water	Drenching	Oral	JZ185	Hm
	*Pentatropis nivalis* (J.F.Gmel.) D.V.Field & J.R.I.Wood (1)	Marena	Mate organ burn	Leave	CL/W	Pounding, rubbing	Drenching and rubbing	Oral, dermal	JZ093	Hm
	*Carissa spinarum* L. (16)	Leda	Skin disease	Leave	CL/W	Pounding by water	Drenching	Oral	JZ086	Hm
Araceae	*Colocasia esculenta* (L.) Schott (33)	Godere	Asthma, nerve	whole part	H/Hg	Cooking	Eating	Oral	JZ062	Hm
Araliaceae	*Schefflera abyssinica* (Hochst. ex A.Rich.) Harms (3)	Koyira	Tooth age, sudden illness	Leaves	T/W	Crushing	Tie on	Dermal	JZ084	Hm
Asparagaceae	*Asparagus setaceus* (Kunth) Jessop (12)	Serite	Uren problem, respiratory infection	Leave, stem	CL/W	Pounding with black cumin seed and mix with water	Drenching, tie-on	Oral, tie on	JZ117	Both
Asteraceae	*Acmella caulirhiza* Delile (41)	Aidamia	Earache, trypanosomiasis	Leaves, flower	H/W	Pounding by water	Ear drop	Areal	JZ063	Both
	*Artemisia absinthium* L. (14)	Natira	Artists, headache, abortion control, anti-parasite	Leave	H/Hg	Crushing and boiling the leave	Drink and sniffing	Oral	JZ101	Both
	*Artemisia annua* L. (30)	Abukee	Headache, Malaria	Leave	H/Hg	Pounding	Sniffing and drenching	Nasal and oral	JZ002	Both
	*Aspilia africana* (Pers.) C.D.Adams (3)	Kishikisha	Nerve, kidney problem	Leave	H/W	Crushing	Chewing	Oral	JZ077	Hm
	*Conyza bonariensis* (L.) Cronquist (1)	Bosha	Common cold	Leave	H/W	Crushing and pounding	Eaten or drenching	Oral	JZ020	Both
	*Crassocephalum macropappum* (Sch. Bip. ex A. Rich.) S. Moore (1)	yegishatele	Wound healing	Flower, leave	H/W	Pounding	Drenching	Oral	JZ149	Both
	*Echinops amplexicaulis* Oliv. (23)	Buris	Fever, evil eye, headache, abdominal pain	Tuber	H/W	Pounding the tuber and mix with water	Drenching	Oral	JZ183	Both
	*Echinops kebericho* Mesfin (27)	Dechmirich/keberich	Cancer, Sudden illness, evil eye	Tuber	H/Hg	Pounding	Fumigate, drink	Oral, dermal	JZ041	Hm
	*Gnaphalium rubriflorum* Hilliard (17)	Zenbano	Headache, fever, hypertension	Leave	H/W	Pounding with the *Ocimum lamifolium*	Sniffing and drenching	Oral	JZ173	Both
	*Solanecio gigas* (Vatke) C. Jeffrey (1)	Olomo	Dysentery	Leaves	S/W	Pounding the leave	Drench	Oral	JZ174	Lv
	*Tagetes minuta* L. (7)	Gemee	Insect repellant	Leaves	H/W	Cutting	Smoking or fumigation	Fumigation	JZ055	Hm
	*Vernonia adoensis* Sch.Bip. ex Walp. (3)	Buza	Wound, skin rushing	Leaves	S/W	Pounding with *Aloe* spp	Smearing or drenching	Oral	JZ026	Hm
	*Vernonia amygdalina* Delile (34)	Gera	Wound, malaria, breast pain, skin disease, evil eye	Leaves	S/W	Pounding with the leave of *Withania somnifera* and* Datura stramonium*	Bathing, drenching	Oral, dermal	JZ056	Both
	*Vernonia cinerascens* Sch.Bip. (5)	Ginagina	Urinary tract infections, male sterility, constipation	Stem, leaves	S/W	Cutting equal sticks and rubbing on the abdomen, pained by hotted, and pounding for internal disease	Rubbing, drenching	Dermal	JZ060	Both
	*Vernonia hochstetteri* Sch.Bip. ex Hochst. (2)	Mono	Wound, abdominal pain	Leaves	S/W	Pounding with *Solanum incanum* and filtering	Drenching	Oral	JZ098	Both
	*Xanthium strumarium* L. (5)	Elahotele	Dandruff, head wound, skin rushes	Leaves	H/FL	Powdering and mixing with butter	Dressing or rubbing	Dermal	JZ046	Both
Balanitaceae	*Balanites aegyptiaca* (L.) Delile (4)	Badena	Tapeworm, toothache	Seed	T/W	crushing and eating the seed	Eating	Oral	JZ016	Hm
Balsaminaceae	*Impatiens rothii* Hook.f. (5)	Wusollua/insosilla	Abortion, wound	Tuber	H/Hg	Pounding	Drenching	Oral	JZ145	Hm
Boraginaceae	*Cordia africana* Lam. (3)	Moha	Urine retention	Leave	T/W	Pounding	Drenching	Oral	JZ097	Hm
	*Ehretia cymosa* Thonn. (1)	Etiwarjii/ulaga	kidney problem	Leave	T/W	Crushing and pounding	Drenching	Oral	JZ052	Hm
Brassicaceae	*Brassica carinata* A.Braun (2)	Santaayfe	Epilepsy	Seed	H/Hg	Roasting the seed and eating it with butter or alone	Drenching	Oral	JZ178	Hm
	*Brassica nigra* (L.) W.D.J.Koch (3)	Senafich	Nerve problems, diarrhea, vomiting, heart disease	Seed	H/Fl	Pounding the seed	Eating	Oral	JZ115	H
	*Lepidium sativum* L. (10)	Sibika/feto	Gastric, thorax disease, colic	Seed	H/W	Crushing and pounding by water	Drenching	Oral	JZ124	Both
Bromeliaceae	*Ananas comosus* (L.) Merr. (16)	Ananas	Heartburn, skin disease	Fruit	H/Fl	Cutting	Eating	Oral	JZ162	Hm
Burseraceae	*Boswellia papyrifera* (Caill.) Hochst. (3)	Eatan zaf	Evil eye, inflammation	Bark	T/W	Crushing and firing	Fumigating	Dermal	JZ045	Hm
Cannaceae	*Canna indica* L (13)	Setakurii	Malaria, gonorrhea, earache	Seed, root, flower	H/W	Pounding	Drenching	Oral	JZ118	Hm
Capparaceae	*Maerua oblongifolia* (Forssk.) A. Rich. (1)	Kundoro	Shivering/Gend	Leave	S/W	Pounding in water	Drenching	Oral	JZ177	Lv
Caricaceae	*Carica papaya* L. / (13	Papaya	Gastric, heartburn, wart	Fruit	S/Fl	Cutting	Eating	Oral	JZ108	Hm
Celastraceae	*Catha edulis* (Vahl) Endl. (11)	Cat	Headache, mental illness, asthma	Leave, stem	S/H	Chewing	Chewing	Oral	JZ028	Hm
	*Hippocratea africana* (Willd.) Loes. ex Engl. (1)	Daniko	Common cold	Leaves	CL/W	Pounding	Fumigation	Oral	JZ039	Hm
Combretaceae	*Combretum molle* R.Br. ex G.Don (4)	Sobo	Fever, constipation, headache, malaria	Bark	T/W	Pounding and mix with *Ocimum lamifolium*	Drenching	Oral	JZ128	Both
Commelinaceae	*Commelina benghalensis* L. (2)	Delisha	Skin disease, wound	Leave, flower	H/W	Pounding by water	Rubbing	Dermal	JZ042	Both
Crasulaceae	*Kalanchoe petitiana* A.Rich. (9)	Korde	Lung fever	Leave, Seed	H/Hg	Pounding	Drenching	Oral	JZ081	Hm
Cucurbitaceae	*Cucurbita pepo* L. (6)	Dubba	Tapeworm, hypertension	Seed, fruit	CL/Hg	Cooking	Eating	Oral	JZ044	Hm
	*Lagenaria siceraria* (Molina) Standl. (2)	Gosee/kill	Infertility, tapeworm	Seed	CL/Hg	Roasting the seed	Eating	Oral	JZ073	Hm
	Momordica foetida Schumach. (3)	Ache	Gastric, hair loss, skin disease	Leave	Cl/W	Chopping	Chewing	Oral	JZ003	Hm
Cupressaceae	*Juniperus procera* Hochst. ex Endl. (17)	Habesha tid	Evil eye, abdominal pain, Pasteurellosis	Leave, Seed	T/W	Squeezing the leave and crushing the cone (for animal salt is added)	Drenching	Oral	JZ137	Lv
Cyperaceae	*Schoenoplectus corymbosus* (Roth ex Roem. & Schult.) J.Raynal (1)	Cecha/cyperus	Wart	Seed	H/W	Pounding with *Nicotiana tabacum* seed	Tie on, drench, or rubbing	Oral, dermal	JZ029	Hm
Dennstaedtiaceae	*Pteridium aquilinum* (L.) Kuhn (9)	Simiza	Cancer, TB, arthritis	Leaves	H/W	Decoction	Bathing, drenching	Oral	JZ126	Hm
Dracaenaceae	*Sansevieria forskaliana* (Schult. & Schult.f.) Hepper & J.R.I. Wood (3)	Qaca	Earache, Malaria	Bulb	H/W	Cutting	Rubbing	Aerial	JZ111	Hm
Ericaceae	*Agarista salicifolia* (Lam.) G.Don (1)	Gaso	Itching	Leave	S/Rs	Grinding	Rubbing	Dermal	JZ176	LV
Euphorbiaceae	*Croton macrostachyus* Hochst. ex Delile (40)	Anka	Bleeding, skin disease, dandruff	Leave	T/W	Squeezing	Smearing, tie on	Dermal	JZ010	Both
	*Euphorbia abyssinica* J.F.Gmel. (1)	Akirsa	Hepatitis	Latex	S/Rs	Cutting and Eating with *Ensete ventricosum* product of Kocho	Eating	Oral	JZ165	Hm
	*Euphorbia ampliphylla* Pax (1)	Argide	Gonorrhea	Leave, Root	S/Rs	Decoction	Drenching	Oral	JZ012	Hm
	*Jatropha curcas* L. (2)	Jatrova	Wound	Latex	S/Rs	Cutting and dropping the mucus	Hold on	Dermal	JZ068	Hm
	*Manihot esculenta* Crantz (26)	Cassava	Hypertension, arthritis	Root	S/Hg	Powdering, cooking	Eating	Oral	JZ027	Hm
	*Ricinus communis* L. (5)	Guloo	Wound, tonsillitis	Fruit, root	S/W	Pounding	Painting or rubbing, drenching	Oral, dermal	JZ064	Hm
	*Sapium ellipticum* (Hochst.) Pax (2)	Wuzingie	Skin disease, digestive problem	Leaves, root	T/W	After decocting the leave or root, it is taken with honey	Drenching and rubbing	Oral, dermal	JZ146	Lv
	*Tragia cinerea* (Pax) M.G.Gilbert & Radcl.-Sm. (3)	Aleblabit	Snake bit	Root	S/W	Pounding	Drenching	Oral	JZ004	Hm
Fabaceae	*Albizia gummifera (J.F.Gmel.) C.A.Sm.* (3)	Sisa	Hypertension, eye disease, diabetes	Seed, root	T/W	Pounding with water	Drenching	Oral	JZ127	Hm
	*Cicer arietinum* L. (2)	Shimbira	Cholesterol, kidney problem	Seed	H/Fl	Powdering	Eating	Oral	JZ120	Hm
	*Erythrina abyssinica* Lam. (1)	Korch	Diarrhea	Root, leave	T/W	Pounding	Drenching	Oral	JZ080	Both
	*Erythrina brucei* Schweinf. (3)	Quora	Snake bit, cancer, Nerve/paralysis	Root, leave	T/W	Pounding	Drenching	Oral	JZ161	Both
	*Millettia ferruginea* (Hochst.) Hochst. ex Baker (2)	Zagie	Skin disease	Leave	T/W	Pounding	Drenching	Oral	JZ153	Hm
	*Parochaetus communis* D. Don (1)	Yemidir koso	Abdominal pain	Leave, seed	H/W	Pounding	Drenching	Oral	JZ150	Hm
	*Pterolobium stellatum* (Forssk.) Brenan (3)	Pinduki	Chickenpox, TB, stomachache	Leaves	CL/W	Chewing for TB, pounding and filtering with food for hen or inject	Chewing, inject	Dermal, oral	JZ109	Both
	*Senna occidentalis* (L.) Link (2)	Shoshainxersa	Snake bit	Leaves	S/W	Pounding	Tie on and Drenching	Oral	JZ122	Hm
	*Tamarindus indica* L. (9)	Roqa	Tape worm, malaria	Seed	T/W	Cutting	Drenching	Oral	JZ113	Hm
	*Tephrosia pumila* (Lam.) Pers. (1)	Charend	Back pain	Leaves	H/W	Pounding	Rubbing and tying on	Dermal	JZ030	Hm
	*Vigna subterranea* (L.) Verdc*.* (5)	Lewz	Digestive problem, sexual impotence	Seed	H/Hg	Roasting the seed and eating it with butter	Eating	Oral	JZ087	Hm
Lamiaceae	*Ajuga integrifolia* Buch.-Ham. ex D.Don (19)	Harmagusa	Diarrhea, eye disease, placenta problem, wound	Leave	H/W	Squeezing	Drenching	Oral	JZ067	Hm
	*Becium grandiflorum* (Lam.) Pic.Serm. (3)	Pitisa	Smallpox (kufign, mich, malaria	Leave	H/W	Crushing and pounding	Bathing and drenching	Dermal, Oral	JZ110	Hm
	*Clerodendrum alatum* Gürke (1)	Alga	Urinary problems	Leave	H/W	Squeezing the juice	Rubbing, drenching	Oral, dermal	JZ005	Lv
	*Clerodendrum myricoides* (Hochst.) R.Br. ex Vatke (7)	Boymech	Hypertension	Leave	S/W	Pounding	Drenching	Oral	JZ022	Hm
	*Coleus abyssinicus* (Fresen.) A.J.Paton (1)	Shona	Gend/shivering disease	Leaves	S/W	Pounding by water and filter	Injection or drenching	Oral	JZ121	Lv
	*Fuerstia africana* T.C.E. Fr. (8)	Yeteja lebeq	Toothage, eye disease, febrile illness	Leaves	H/W	Squeezing	Drenching and creaming	Oral, dermal	JZ152	Hm
	*Leonotis ocymifolia* (Burm.f.) Iwarsson (3)	kata lush	Headache, depression	Leave	S/W	Crushing	Sniffing	Nasal	JZ071	Hm
	*Leucas tomentosa* Gürke (3)	Daracha	Headache, skin rashes, fever	Leave	H/W	Pounding	Drink and rub	Oral, dermal	JZ172	Both
	*Mentha spica* L. (23)	Sheyketal	Headache, common cold	Leave	H/Hg	Boiling the leave and drink	Sniffing/ inhaling the vapor as fumigate	Dermal	JZ100	Hm
	*Ocimum basilicum* L. (13)	Besobila	Digestive problem	Leave, seed	H/Hg	Cooking	Eating	Oral	JZ017	Hm
	*Ocimum lamiifolium* Hochst. ex Benth. (56)	Mechtale	Febrile illness, headache, skin disease	Leave	S/Hg	Decocting the leave alone	Drink and rubbing	Oral	JZ019	Hm
	*Ocimum urticifolium* Benth. (31)	Damakase/anchaf	Asthma, headache, febrile illness, mastitis	Leave	S/W	Pounded and drenched the filter with salt	Sniffing and drenching	Nasal, oral	JZ036	Both
	*Rosmarinus officinalis* L. (22)	Sigametbesha	Headache, depression, asthma	Leaves	S/Hg	Pounding and boiling with coffee and *Ruta chalepensis* leaves or seed	Drenching, sniffing	Nasal, oral	JZ125	Hm
	*Salvia nilotica* Juss. ex Jacq. (9)	Gasind/deladhae	Wound, maceration	Leaves	H/W	Pounding	Drenching	Oral, dermal	JZ054	Both
	*Satureja* genus (6)	Yesukar medanit	Hypertension, high cholesterol	Leaves	S/Hg	Crushing	Chewing	Oral	JZ021	Hm
	*Thymus vulgaris* L. (3)	Tosign	Headache, asthma	Leaves	H/Hg	Crushing and pounding	Sniff and smoke	Nasal	JZ139	Hm
Lauraceae	*Persea americana* Mill. (19)	Avocado	Skin disease, gastric, diabetes	Fruit	S/Hg	Cutting the fruits and eat	Eat	Oral	JZ015	Hm
Linaceae	*Linum usitatissimum* L. (5)	Telba	Dry common cold, gastric	Seed	H/Hg	Powdering and mixing with *Eragrostis tef*	Drenching	Oral	JZ133	Hm
Loranthaceae	*Phragmanthera regularis* (Steud. ex Sprague) M.G.Gilbert (2)	Tsensa	Intestinal infection	Leaves	Ep/W	Pounding	Drenching	Oral	JZ141	Hm
Malvaceae	*Dombeya torrida* (J.F.Gmel.) Bamps (1)	Lolashe	Wound	Root	T/W	Powdering	Dressing	Dermal	JZ182	Both
	*Gossypium hirsutum* L. (2)	Tit	Bleeding, wound	Seed	S/Fl	Cutting	Hold on	Dermal	JZ035	Both
	*Sida ovata* Forssk. (3)	Chursa	Skin infections, stomachache, thorax	Leaves	S/W	Grinding and pounding	Rubbing, washing	Dermal	JZ033	Hm
	*Sida rhombifolia* L. (1)	Danderuta	Constipation	Leaves	H/W	Pounding the leave and mix with butter	Drenching	Oral	JZ038	Both
	*Sida schimperiana* Hochst. ex A.Rich. (2)	Chifrig	Wound, fever	Root	H/W	After washing the root, it pounded	Hold on	Dermal	JZ032	Hm
Meliaceae	*Ekebergia capensis* Sparrm. (1)	Ononu	Cancer	Bark	T/W	Pounding	Drenching	Oral	JZ107	Hm
	*Melia azedarach* L. (3)	Nime	Skin disease, toothache, dandruff	Leave	T/W	Chewing	Chewing	Oral	JZ170	Both
Melianthaceae	*Bersama abyssinica* Fresen. (1)	Azamir	Rabies	Root	S/W	Pounding	Drenching	Oral	JZ160	Hm
Menispermaceae	*Stephania abyssinica* (Quart. -Dill. & A. Rich.) Walp. (2)	Kelala	Wound, vomiting	Root	CL/W	Pounding with *Argemone mexicana*	Hold on or tie on	Dermal	JZ074	Both
Moraceae	*Ficus sur* Forssk (2)	Ase/shola	Heart problem, wound	Bark	T/W	Pounding, dressing	Rubbing	Oral, dermal	JZ184	Hm
	*Ficus sycomorus* L. (2)	Maro	Stomach-ache	Leaves	T/W	Pounding with water	Drenching	Oral	JZ094	Hm
	*Morus alba* L. (2)	Enjorii	Nerve problem, headache	Leave, fruit	CL/W	Pound the leave and mix with butter, or cut and eat fruit directly	Drink the juice and eat the seeds	Oral	JZ047	Hm
Moringaceae	*Moringa stenopetala* (Baker f.) Cufod. (51)	Alleko	Malaria, kidney problem	whole part	T/W	Grinding or pounding	Drenching	Oral and dermal	JZ007	Hm
Musaceae	*Ensete ventricosum* (Welw.) Cheesman (30)	Enset	Gastric, hypertension	whole part	S/Hg	Cooking or using as food	Eating	Oral	JZ049	Both
Myrtaceae	*Eucalyptus citriodora* Hook. (29)	Sheto beharzaf	Headache, fever, common cold, thorax	Leave, bark	T/Fl	Crushing and firing the leave	Inhale smoke	Fumigation	JZ104	Hm
	*Eucalyptus globulus* Labill. (20)	Nech beharzaf	Headache, febrile illness, cough, urine burn, black leg	Leave	T/W	Fire the leave and inhale the smoke, and pounding with salt for animal	Fumigation, washing	Dermal	JZ102	Both
	*Psidium guajava* L. (5)	Zeytun	Gastric, infertility	Fruit	S/Hg	Cutting and eating fruits more	Eating	Oral	JZ164	Hm
	*Syzygium guineense* (Willd.) DC. (2)	Oche	Abdominal pain	Fruit, bark	T/W	Cutting	Eating	Oral	JZ105	Hm
Olacaceae	*Ximenia americana* L. (4)	Enkoy	Fever, toothache, common cold	Fruit	T/W	Cutting the fruits and eat	Eat	Oral, dermal	JZ148	Hm
Oleaceae	*Jasminum grandiflorum* L. (23)	Tembelel/deda tura	Hepatitis, earache, cough, sexual impotence, headache, skin disease	Leaves, flower	CL/W	Decoction, pounding, and powdering	Rubbing, washing, dropping, drink	Oral, dermal, nasal, optical	JZ163	Hm
	*Olea europaea* L. (13)	Weyra	Heart disease, malaria, stomachache	Leave, seed	S/W	Pounding the leave	Drenching	Oral	JZ144	Hm
	*Olea welwitschii* (Knobl.) Gilg & G.Schellenb. (1)	Damot weyra	Toothage	Stem, Leaves	S/W	Cutting and brushing by the sticks or leaves	hold on and rubbing	Oral	JZ037	Hm
Oxlidaceae	*Oxalis corniculata* L. (1)	Zelamata	Wound	Leave	H/W	Crushing and tying on	Tie on	Dermal	JZ154	Hm
Papaveraceae	*Argemone mexicana* L. (5)	Nech lebash	Wound	Stem	H/W	Cutting and dropping the mucus on the wound	Drop on	Dermal	JZ103	Both
Peraceae	*Clutia abyssinica* Jaub. & Spach (3)	Feyelfej	Infertility, malaria, gonorrhea	Root, leaves	S/W	Pounding	Drenching	Oral	JZ034	Both
	*Clutia lanceolata Forssk.* (4)	Fyle feji	Diabetes	Leave	S/W	Pounding	Drenching	Oral	JZ171	Hm
Phyllanthaceae	*Bridelia scleroneura* Müll.Arg*.* (1)	Zuzie	Inflammation	Leave, stem	T/W	Pounding with water	Drenching	Oral	JZ157	Lv
Phytolacaceae	*Phytolacca dodecandra* L'Hér. (32)	Anchiche	Skin infection	Leaves	CL/W	Pounding	Rubbing	Dermal	JZ009	Hm
Plumbaginaceae	*Plumbago zeylanica* L. (4)	Amira	Eye disease, toothache, earache	Leaves, fruit	H/W	Squeezing and pounding	Dropping ear, drenching	Oral, Aerial	JZ008	Hm
Poaceae	*Cymbopogon citratus* (DC.) Stapf (6)	Frenji tejisar	Headache, blackleg, insect repellant	Leave	H/Hg	Crushing and boiling	Drenching	Oral	JZ158	Both
	*Cymbopogon martini* (Roxb.) Will.Watson (3)	Tejisar	Intestinal infection, headache	Leave	H/Hg	Boiling the leave and drinking tea	Drenching	Oral	JZ132	Both
	*Eragrostis tef* (Zuccagni) Trotter (2)	Tef	Heart problem	Seed	H/Fl	Cooking the powder	Eating	Oral	JZ076	Hm
	*Panicum maximum* Jacq. (1)	Tura	Bleeding	Leave	CL/W	Pounding	Tie on	Dermal	JZ143	Both
	*Pennisetum sphacelatum* (Nees) T.Durand & Schinz (2)	Sendedo	Wound, eye disease	Leaves	H/W	Pounding, rubbing	Holding on and rubbing	Dermal	JZ116	Both
	*Saccharum officinarum* L. (5)	Sugarcane	Gastric, hepatitis	Stem	S/Hg	Cutting the stem and chewing	Eating	Oral	JZ130	Both
Podocarpaceae	*Podocarpus falcatus* (Thunb.) Endl. (5)	Zigba	Tapeworm, eye disease, malaria	Seed	T/W	Pounding and powdering	Drenching and rubbering	Optical, oral	JZ155	Both
Polygalaceae	*Securidaca longepedunculata* Fresen. (5)	Sangana	Headache, stomach-ache, TB, Inflammation	Leave, root, bark	S/W	Crushing and powdering	Drenching	Oral	JZ114	Both
Polygonaceae	*Rumex abyssinicus* Jacq. (19)	Meqmeko	Diabetes, cancer	Leaves, root	S/W	Chewing or filter after pounding, then mix with honey	Drenching	Oral	JZ095	Hm
	*Rumex nepalensis* Spreng. (2)	Qtel rejim	Uterine bleeding	Root, leaves	H/W	Chewing	Eating	Oral	JZ112	Both
	*Drynaria volkensii* Hieron.(1)	Kakiyee	Wound	Leave	EP/W	Squeezing	Tinning on	Dermal	JZ069	Hm
Primulaceae	*Embelia schimperi* Vatke (2)	Enkoko	Gastric	whole part	CL/W	Pounding and chopping	chew and drink	Oral	JZ048	Hm
	*Maesa lanceolata* Forssk*.* (3)	Terika	Skin infection, Gend/shivering disease	Leave	S/W	Pounding and filtering	Drenching or injecting	Oral	JZ140	Both
Ranunculaceae	*Clematis hirsuta* Perr. & Guill. (1)	Soge	Wound	Leave	CL/W	Pounding with water	Drenching	Oral	JZ129	both
	*Nigella sativa* L. (31)	Tikur azmud	Headache, febrile illness, athletic feet, skin infection, tonsilitis	Seed	H/Hg	Pounding, powdering	Sniffing, dropping, rubbing	Aerials, dermal, oral	JZ013	Hm
Rhamnaceae	*Rhamnus prinoides* L'Hér. (3)	Gesho	Blood vessel problem, ringworm	Leaves, seed	S/Hg	Grind and pound by water and filter	Drenching	Oral	JZ057	Both
	*Ziziphus mauritiana* Lam. (3)	Kurkura	Hepatitis, abdominal pain	Seed	T/W	Cutting	Eat	Oral	JZ085	Hm
Rosaceae	*Hagenia abyssinica* (Bruce) J.F.Gmel. (35)	Koso	Tapeworm	Seed	T/W	Pounding	Drenching	Oral	JZ083	Hm
	*Malus sylvestris* (L.) Mill. (3)	Apple	Gastric, cancer, inflammation	Fruit	T/Hg	Cutting	Eating	Oral	JZ011	Hm
	*Prunus africana* (Hook.f.) Kalkman (3)	Okanse	Gonorrhea, malaria	Bark, root	T/W	Pounded and filtered, mix with butter or drink with honey	Drenching	Oral	JZ106	Both
Rubiaceae	*Canthium oligocarpum* Hiern (3)	Galo	Headache, internal infection, skin disease	Leave	S/W	Pounding	Drenching	Oral	JZ188	Hm
	*Coffea arabica* L. (27)	Buna	Wound, depression, headache	whole part	S/Hg	Roasting and powdering	Drenching	Oral	JZ024	Both
	*Galiniera saxifraga* (Hochst.) Bridson (2)	Darume	Bone rupture	Stem, Leave	S/W	Crushing and mixing with water	Drenching	Oral	JZ180	Both
	*Mussaenda arcuata* Poir. (1)	Munmuno	Earache	Leave	CL/W	Squeezing and filter	Drop on	Aerial	JZ099	Hm
	*Pavetta abyssinica* Fresen. (2)	Dingay seber	Wound, inflammation	Leaves	S/W	Pounding, powdering	Painting, drenching	Oral, dermal	JZ187	Both
	*Pentas lanceolata* (Forssk.) Deflers (1)	Maratale	Bone rupture	Leaves	S/W	Pounding and mix with salt	Tie on	Dermal	JZ092	Lv
	*Pentas schimperiana* Vatke (4	Darense, waynagift	Urine problems, earaches, eye disease	Leaves, flower	H/W	Powdering	Rubbing, dropping on	Dermal	JZ040	Hm
	*Psydrax schimperiana* (A. Rich) Bridson subsp. (3)	Gila	Febrile illness, cancer	Leaves, seed	S/W	Powdering	Rubbing	Oral	JZ059	Hm
Rutaceae	*Casimiroa edulis* La Llave (9)	Kasmir	Diarrhea	Fruit	T/H	Cutting the fruit and eat	Eating	Oral	JZ070	Hm
	*Citrus* × *aurantiifolia* (Christm.) Swingle (23)	Habesha lomii	Gastric, dandruff	Fruit	S/Hg	Squeezing the juice and mix with a small amount of salt	Drenching	Oral	JZ066	Both
	*Citrus* × *limon* (L.) Osbeck (10)	yefernj lomi	Dandruff, cough	Fruit	S/Hg	Sucking the juice	Eating	Oral	JZ088	Hm
	*Citrus* × *sinensis* (L.) Osbeck (4)	Burtukan	Hypertension, gastric, diarrhea	Fruit	S/Hg	Sucking out the juice	Drenching	Oral	JZ025	Hm
	*Clausena anisata* (Willd.) Hook.f. ex Benth. (3)	Eshima/chuqutiya	Heart failure, kidney, toothache	Leave	S/W	Pounding	Drench, Brushing	Oral	JZ051	Hm
	*Ruta chalepensis* L. (25)	Tselote	Evil eye, asthma, headache, febrile illness, dry cough	whole part	S/Hg	Chopping, crushing the stem, and boiling the leave or pounding the seed	Sniff, fumigate, and drench	Nasal, dermal, oral	JZ136	Both
	*Vepris dainellii* (Pic.Serm.) Kokwaro (1)	Chawla	Vomiting/food poison	Leaves	T/W	Pounding	Drenching	Oral	JZ031	Hm
Sapindaceae	*Dodonaea angustifolia* L.f*.* (3)	Ktkita	Diabetics, infertility, hypertension	Leave, Root	S/W	Pounding	Drenching	Oral	JZ078	Hm
	*Paullinia pinnata* L. / (1)	Tinchro	Skin disease	Leaves	Cl/W	Powdering	Rubbing	Dermal	JZ138	Hm
Scrophulariaceae	*Verbascum sinaiticum* Benth. (2)	yeahiyajero	Earache, wound	Seed, leaves	H/W	Dried, crushed, and powdered	Dropping on	Areal dermal	JZ065	Hm
Simaroubaceae	*Brucea antidysenterica* J.F.Mill. (3)	Shrushika	Bleeding, wound	Leave	S/W	Pounding by water	Drenching	Oral	JZ123	Hm
Solanaceae	*Datura stramonium* L. (4)	Atefaris	Skin disease, toothache, evil eye, head wound	Leave, Seed	H/Rs	Pounding and mixing with *Withania somnifera*	Bathing, rubbing, holding on	Dermal	JZ014	Hm
	*Lycopersicon esculentum* Mill. (1)	Timatim	Cancer	Fruit	H/Fl	Cooking	Eating	Oral	JZ159	Both
	*Nicotiana tabacum* L. (36)	Tembaho	Headache, depression, asthma, cough	Leave	H/W	Grind and crushing	Sniffing fumigates	Fumigations	JZ135	Hm
	*Physalis peruviana* L. (12)	Birike	Constipation, cancer, kidney problems, hepatitis	Leaves	H/W	Pounding	Drenching	Oral	JZ018	Hm
	*Solanum incanum* L. (9)	Buloo	Abdominal pain, tonsilitis	Root, leave, seed	S/W	Pounding	Drink, hold on	Oral	JZ023	Hm
	*Solanum tuberosum* L. (2)	Sweet potato	Cancer, ulcers	Tuber	H/Fl	Cooking	Eating	Oral	JZ131	Hm
	*Withania somnifera* (L.) Dunal (39)	Gezawa	Typhoid, evil eye, asthma, wound, skin disease, trypanosomiasis	whole part	S/W	Pounding by water with the leaves of *Thymus schimperi* and *Datura stramonium and filter*	Drink, bathing	Oral, dermal	JZ058	Hm
Thymelaceae	*Gnidia stenophylla* Gilg (3)	Tumano	Malaria	Bark	H/W	Pounding	Drenching	Oral	JZ142	Hm
Urticaceae	*Urera hypselodendron* (Hochst. ex A. Rich.) Wedd. (3)	Halilo	Constipation, inflammation	Leaves	S/W	Pounding the leave and mix with salt	Drenching	Dermal, oral	JZ175	Lv
	*Urtica dioica* L. (3)	Pudo	Urine problem, itching	Leaves	H/W	Pounding and rubbing	Drenching	Oral	JZ166	Both
	*Urtica simensis* Hochst. ex A.Rich. (5)	Sama	Heart problem	Leaves	H/W	Cooking the leave and eat	Eating	Oral	JZ167	Hm
Verbenaceae	*Lantana trifolia* L. / (5)	Mirimich	Gastric, bleeding, toothache	Leave	S/W	Pounding and holding on	Drench, hold on	Oral	JZ096	Hm
	*Lippia adoensis* Hochst. ex Walp. (2)	Shasha	Breast cancer, cough	Leave	S/W	Pounding, smoking	Fumigates, drenching	Oral	JZ119	Hm
Vitaceae	*Cyphostemma adenocaule* (Steud. ex A.Rich.) Desc. ex Wild & R.B.Drumm*.*(1)	Jeljelo	Wound	Leave	CL/W	Decocting	Tie on	Dermal	JZ181	Both
Zingiberaceae	*Aframomum corrorima* (A.Braun) P.C.M.Jansen (15)	Korarima	Heart pain, blood circulation, Lung fever	Seed	H/Hg	Pounding and boil with *Allium sativum*	Drenching	Oral	JZ079	Hm
	*Curcuma longa* L. (9)	Erd	Stroke, diabetes, hepatitis, cancer	Rhizome	H/Hg	Pounding with the leave of *Allium sativum*	Drenching, eating	Oral	JZ050	Hm
	*Zingiber officinale* Roscoe (21)	Zingible	Bronchitis, tonsil, heart case, wart	Tuber	H/Hg	Pounding with *Withania somnifera* leave	Chewing, powdering	Oral	JZ156	Hm

*PU* part used, *Ht* habits, *Hbt* habitats, *MA* mode of application, *RA* routes of administration, *V. N* voucher number, *S* shrubs, *H* herbs, *T* trees, *CL* climbers, *EP* epiphyte, *W* wilds, *Hg* home Garden, *RS* roadside, *FL* farmland, *Hm* human, *Lv* livestock

*Ocimum lamiifolium* contains flavonoids, tannins, and saponins, and its oil is the primary source of linalool [40, 41]. Furthermore, *Artemisia* species, part of the Asteraceae family, are widely grown in the region and commonly utilized for cultural and commercial purposes. They contain various beneficial compounds, including disaccharides, polysaccharides, glycosides, saponins, terpenoids, flavonoids, carotenoids, and essential oils. These plants have significant biological importance, serving as antiparasitic, anti-malarial, antihyperlipidemic, antiasthmatic, antiepileptic, antitubercular, antihypertensive, antidiabetic, antiemetic anxiolytic, antidepressant, anticancer, hepatoprotective, gastroprotective, insecticidal, antiviral agents, and even against COVID-19 [42]. An important compound extracted from species of *Artemisia*, Artemisinin, is used to produce drugs for treating malaria and viral diseases [43, 44]. In-depth explorations, phytochemical isolation, and characterization of species from those prominent families are essential for novel drug discovery [45, 46].

**Fig. 3:**
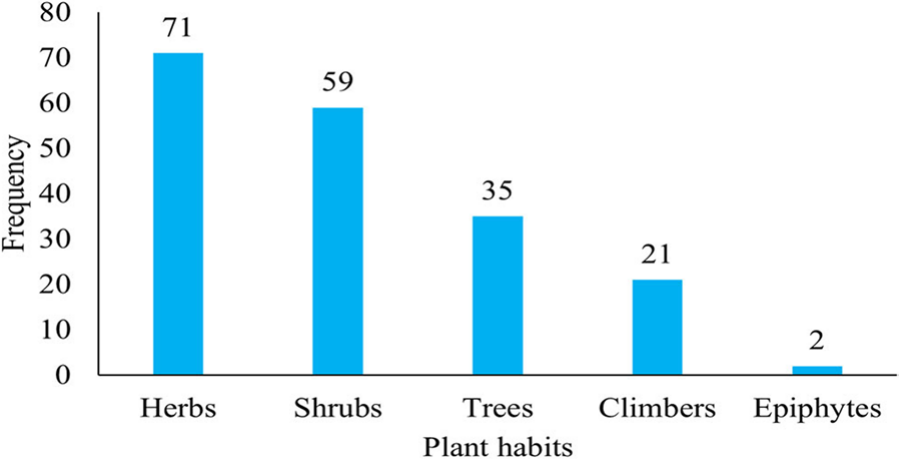
Habits of recorded medicinal plants

Herbalists in the study area mainly use herbaceous plants, with 71 species, followed by shrubs, trees, climbers, and epiphytes with 59, 35, 21, and 2 species, respectively (as shown in Fig. 3). Herbaceous species are primarily available during the rainy season, while some herbalists collect a few plants in their home gardens. Some species are harvested during the rainy season and kept for use in drier times. For instance, species such as *Acmella caulirhiza*, *Echinops amplexicaulis*, *Gnaphalium rubriflorum*, *Gnidia stenophylla*, and *Pennisetum sphacelatum* were harvested during the rainy season and stored for use in the dry season. *Thunbergia abyssinica* and *Impatiens rothii* are among endemic species found to have a high distribution in the area. *Echinops amplexicaulis* was found to be distributed widely and is a vital herbal species in the region. However, moss and liverwort were not encountered in the study, most likely because they grow during the rainy season. Healers confirmed that they could not usually assess those species frequently in the dry season. In Ada’a district, in other parts of the country, most people utilize shrubs for traditional medicine [47]. However, the present findings support herbal extraction of Ethiopian medicinal plants, which has attracted interest of researchers due to their potential sources of active compounds that benefit against several diseases and play a critical role in meeting society's basic medical demands. Hence, they are significant sources of medicine for the local communities [33, 48, 49].

The present study documented higher number of medicinal plants species as compared to other similar studies conducted elsewhere in Ethiopia with a range of ethnic groups. This comparison is important to overview the Ethnolinguistics diversity and availability of medicinal plants. Further it highlights promising therapeutic medicinal plant species used for drug discovery [46]. Among others, a study conducted on medicinal plants used in Loma and Gena Bosa Districts of Dawro Zone, documented about 178 species, southern Ethiopia [50]. Fisseha Mesfin (2009) documented 198 plant species in Wonago Woreda with Gedeo community, Southern Ethiopia [51]. Similarly, Endalew Amenu (2007) documented a total of 188 plant species with indigenous people of Ejaji area, Chelya District, west Shewa in Ethiopia, and the highest informant consensus was recorded for *Ocimum urticfoluim* in treating febrile illness in the area [52]. Moa Megersa (2010) documented 126 MPs for their medicinal uses in Wayu Tuka Woreda, East wollega Zone of Oromia Region, Ethiopia, found *Acmella caulirhiza* was the most preferred medicinal plant by local people of the study area to treat tonsillitis [53]. Similarly, the Afar people in Chifra have reported the healing potential of *Aloe* spp for malaria [54]. In the Wonago area, *Artemisia afra* mainly used for headache treatment [51]. The Sheko people in southwestern part of the country uses *Ocimum lamiifolium* and *Phytolacca dodecandra*, to treat skin and gastrointestinal diseases[51]. *Zingiber officinale* for tonsillitis, *Clerodendrum myricoides* for tumor, *Hagenia abyssinica* for tapeworm, *Ricinus communis* for rabies, *Prunus africana* for wound healing around Bale Mountain [25]. The presence of above-mentioned species in present study may indicate the healing potential of those medicinal plants and importance of local vegetation in the present study area for traditional medicine reserves. The present study reports on the wide usage of various new species, such as *Acanthus sennii*, *Gnaphalium rubriflorum*, *Gnidia stenophylla*, *Impatiens rothii*, *Olea welwitschia*, *Pennisetum sphacelatum*, *Solanecio gigas* and *Thunbergia abyssinica*. These findings contribute new plant uses to the field of Ethnopharmacology in the country (Table 3).

**Fig. 4:**
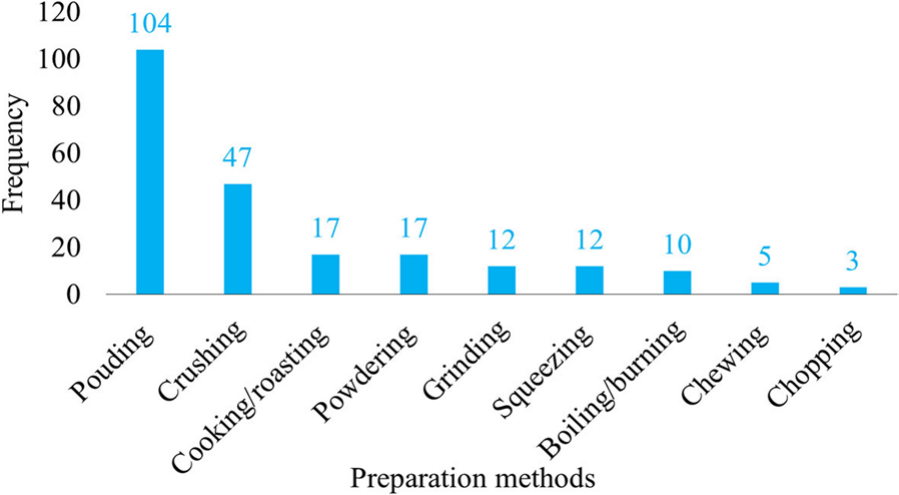
Preparation methods of ethnomedicine

**Fig. 5:**
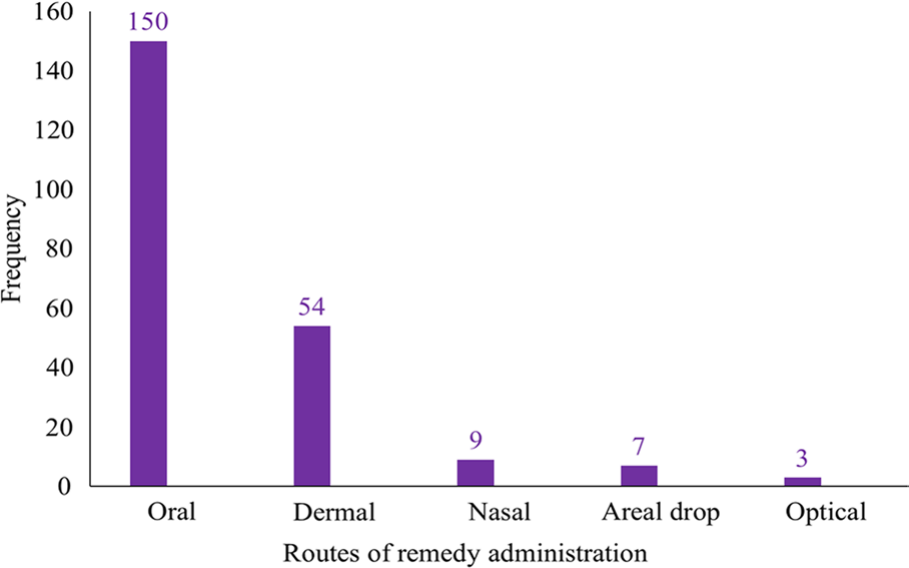
Ethnomedicines application routes

**Fig. 6:**
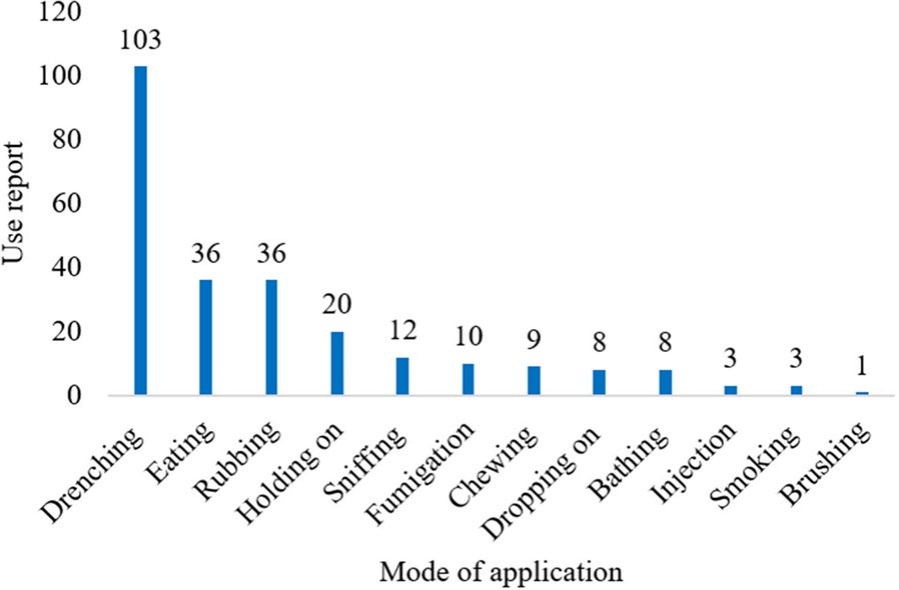
Ethnomedicines application modes

The relatively high availability of herbaceous medicinal plants in comparison to other plant habits could account for their widespread use in different corners of the country [55, 56]. They adapted to wide environmental ecology and exhibit major plant habits [15]. *Acanthus sennii*, *Echinops kebericho*, *Ensete ventricosum*, *Erythrina brucei*, *Impatiens rothii*, *Kalanchoe petitiana*, *Lippia adoensis*, *Millettia ferruginea*, *Solanecio gigas*, *Urtica simensis* and *Vepris dainellii* are some of the widely distributed endemic medicinal plants in the area (Figs. 4, 5 and 6).

Wild forest is the main source of Gamo medicinal plants, accounting for 128 species. Others are collected from home gardens (43 species), farmlands (11 species), and roadside (6 species). This finding coincided with similar study results conducted elsewhere [38, 48, 49, 57–59]. Healers keep track of the best locations for different species of plants in the forest. They also help researchers obtain unbiased data from local healers while ensuring that they maintain strict confidentiality about cultural beliefs and practices. This information is valuable in studying medicinal plants [32, 36]. However, conservation efforts to preserve these plants are limited in the area, even though the natural forest is rapidly disappearing due to various factors like deforestation [60]. This is concerning because the loss of forest environments translates to the loss of valuable traditional knowledge about medicinal plants [33]. The local vegetation has ever green riparian and swamp forest, small leaved deciduous woodland in hill mountain of Ganta, and at higher altitudes it is possible to observe patches of Bamboo species [15].

### Ethnomedicine preparations and plant part used

Ethnomedicine practitioners of Gamo people use basic techniques and locally available materials, like mortars and pestles, to prepare remedies. A recent study has identified nine major preparation methods, with pounding (104 mentions) and crushing (47 mentions) being the most used. Other methods include cooking (roasting), powdering, grinding, squeezing, boiling, burning, chewing, and chopping, although these were mentioned less frequently. Gamo herbalists use wooden mortars and pestles, known locally as "Mukacha," to easily pound or crush plant parts with common ingredients like honey, coffee, salt, and butter. These additions serve to improve the nutrition and flavor of the remedy for the patient. For instance, a similar practice has been reported in the traditional medicine of the Maonan people in China, where locals add ingredients such as honey, butter, and meat to enhance the nutrition and flavor of the remedies they prepare [61]. Further herbalists merge multiple species to enhance the healing potential of remedies. The present study has identified 12 species of plants that exhibit synergistic effects when used in combination to prepare remedies (Table 3). This discovery is significant as it allows for the development of more effective and targeted treatments for various ailments and health conditions. Among others, *Allium sativum*, *Argemone mexicana*, *Datura stramonium*, *Ensete ventricosum*, *Ocimum lamifolium*, *Ruta chalepensis*, *Solanum incanum*, *Thymus schimperi*, *Trigonella foenum-graecum*, *Withania somnifera* and *Zingiber officinale* are frequently mentioned for their synergic therapeutic use. This traditional practice needs to be supported by scientific studies to overview whether the combination has negative or positive effect, but it clearly shows local herbalists merge more species to prepare effective remedies for fast recovery of patients.

As shown in Table 4 below, about 50% of Gamo traditional medicine was mainly prepared from leaves (119 mentions, 93 species), followed by seeds (33 mentions, 26 species). Other plant components like fruits, bark, stem, tuber, latex, whole portion, flower, and bulbs were also used by Gamo healers in their ethnomedicinal preparation. A similar finding was reported by informants of Kafficho and Sheko people in the southwest part of the country, revealing that leaves and seeds were widely used plant parts, showing the ethnolinguistic interaction of different people and effective concentration of bioactive components present [30, 31]. The cultural, traditional interaction might link ethnobotanical knowledge transfer among various people. However, the present finding contradicts the results of a study conducted elsewhere in the Wonago area, where roots were found to be the most used plant component [37].

Gamo practitioners collect various plant components for remedy preparation under different use conditions. Out of 188 species, 145 species were used in fresh form, 21 in dry form, and 22 in both forms. They prefer to prepare remedies in a fresh form as patients come, and most herbaceous species are usually used freshly. However, when a plant is not easily accessible, herbalists prepare it in dry form, believing it retains its therapeutic properties for a longer time. For instance, a Gamo remedy made from *Nicotiana tabacum* to alleviate asthma, headaches, and coughs is prepared in dry powder form for long-term use (up to a year). Several studies suggested the preference of fresh plant parts by local herbalists [30, 33, 62] which implies that healers regularly collect plant parts [63].

It is a well-established fact that overharvesting has serious consequences for the survival of medicinal plants [64–66]. Despite this, the herbalists of Gamo have developed an effective solution by carefully tending to the mother plants during collection due to their traditional cultural law known as "Wagas and Dubusha," which stems from the belief that everything is connected and bound in a delicate balance. It dictates everything from interpersonal relationships to the conservation and preservation of pasture, forest, soil, and water, because in Wagas all are interconnected, if any one aspect is denied or imbalanced then the whole system is understood to be at risk. This reflects a cultural value that emphasizes sustainable usage and environmental harmony and plays a vital role in preserving local biodiversity [14]. Comparatively, using leaves affects the species' lifecycle less than other parts like root and bark. But some species, such as *Echinops kebericho*, *Embelia schimperi*, *Hagenia abyssinica*, *Moringa stenopetala*, and *Withania somnifera*, have been targeted for their roots or whole plant parts, resulting in possible extinction of those species in the area. Many medicinal plants are overharvested, which puts them at risk of becoming a threatened species. One such example is *Taverniera abyssinica* A. Rich, whose slender roots are wrapped in small, coiled bundles and sold as medicine. The species is not encountered in the present study. This species is currently threatened, and less data are available in Ethiopia [11].

### Route of administration, application mode, and dosage determination

The current study recorded five primary routes of remedy administration—oral (via mouth), dermal (external), nasal, optical, aerial drop, or injection. Gamo healers prefer to use drenching (10 mentioned), rubbing (36 mentioned), or directly eating plant parts (36 mentioned) to treat illnesses. They carefully consider the patient's condition, sex, age, disease type, and other factors to choose the best route, method of application, and dosage. Gamo healers do not recommend oral use of herbal remedies for children and pregnant women (25 mentioned) due to dosage problems [33]. A few other informants (7 mentions) prefer external treatments for children to minimize risks. Similarly, Sheko people healers suggest that a taenicide prepared from fruits of *Embelia schimperi* should not be given to children under 15 due to its adverse effect [30], indicating shared traditional practices.

Herbalists employ various methods to accelerate the healing process, and some of these methods involve multiple routes. In Gamo, herbalists adopt certain precautions for patients, such as refraining from food and drink and spending the morning alone without ingesting food, to enhance the efficacy of remedies. For instance, in the treatment of tapeworm disease, the herbalist prepares a remedy from the *Hagenia abyssinica* species, which the patient takes before breakfast and then fasts for a prolonged period, typically six hours, to expel the worms from the intestine effectively. Similarly, for febrile illness, or locally called Mech, the herbalist fumigates the patient with smoke from the *Ocimum lamiifolium* and *Eucalyptus citriodora* species and advises the patient to sleep as soon as possible. The herbalist makes a diagnosis by conducting visual observations of the patient's eye and skin color, tongue and throat regions, and body temperature while also inquiring about the patient's symptoms. This approach is consistent with the findings of a range of ethnobotanical studies carried out elsewhere in different regions of the country [38, 57].

Traditional healers use simple and often unconventional techniques to determine the appropriate dosage for their patients. For instance, in some cultures, healers use finger strips, glasses, coffee cups, or teaspoons to measure the dosage, depending on the age and sex of the patient. However, the lack of a standardized dosage poses a challenge to the safety and efficacy of traditional medicine.

In some communities like the Gamo people of Ethiopia, healers order different amounts of dosage based on the patient's age, sex, and physical condition. They also use various inputs such as milk, coffee, honey, meat, and "Tella" (a local beer) to reduce the side effects of ailments. These practices reflect the cultural diversity and richness of traditional medicine but also underscore the need for standardization. Dosage and safety are shared problems among the traditional practitioners of the country due to the lack of a solid standard for traditional medicine. It is essential to develop a regulatory framework that considers the unique cultural practices and knowledge of traditional healers while ensuring the safety and efficacy of their treatments [32, 51, 67] (Table 4).

**Table 4:** Plant part used in remedy preparation

Parts used	Citation/mention	Percentage %	Number of species
Leaves	119	50	93
Seeds	33	14	26
Roots	23	10	18
Fruits	20	8	16
Bark	9	4	7
Stems	9	4	7
Flowers	7	3	5
Whole plant parts	7	3	5
Tuber	5	2	4
Bulbs	4	1	3
Latex	3	1	2
Rhizome	1	0	1

### Treated disease type and the healing potential of medicinal plants

Gamo healers used medicinal plants to treat around 80 different disease conditions, grouped into nine broad categories, as shown in Table 5. The sudden sickness disease category received the highest ICF value (ICF: 0.35), followed by blood and circulatory-related disease diseases category (ICF: 0.33). While the lowest ICF values were observed for malaria (Ur: 17), rabies (Ur: 1), snake bite (Ur: 3), Gend/shivering (Ur: 3), insect bite (Ur: 1), disease categories (ICF: 0), and gastrointestinal-related disease (ICF: 0.12). Dermal diseases categories received the highest number of use reports (Nur: 76) and plant species used (61). In contrast, genitourinary system diseases categories including STDs had the lowest value of use reports (Nur: 25) and plant taxa (Nt: 21). The ICF result analysis indicates that the Gamo community employs a variety of plant species to cure specific ailments, underscoring the significance of the region's diverse ecosystem. Moreover, the heterogeneity of medicinal plants used by the Gamo people is a testament to their extensive knowledge of traditional medicine and their ability to interact with natural phenomena to identify essential plants.

**Table 5:** Informant consensus factors (ICF) value of disease categories

Disease categories	Nur	Nt	ICF
Dermal: ((skin-related (Ur:47) and wound-related (Ur:29) disease)	76	61	0.2
Sudden sickness: (headache (Ur: 27), evil eye (Ur: 8), febrile illness (Ur: 8), fever (Ur: 9), inflammation (Ur: 6), sudden illness (Ur: 2)	61	40	0.35
Sensorial organ-related disease: (earache (Ur: 8), eye disease (Ur; 8), toothache (Ur: 11)	27	22	0.19
Malaria (Ur: 17), rabies (Ur: 1), snake bite (Ur: 3), Gend/shivering (Ur: 3), insect bite (Ur: 1)	26	26	0
Respiratory-related disorders: (asthma (Ur: 9), bronchitis (Ur: 1), common cold (ur:7), cough (Ur: 6), chicken pox (Ur: 1), lung fever (Ur: 2), pasteurellosis (Ur: 1), pneumonia (Ur: 1), respiratory infection (Ur: 2), thorax disease (Ur: 2, tonsilitis (Ur: 4)	36	31	0.14
Gastrointestinal-related disease: (abdominal pain (ur:7), diarrhea (Ur: 6), dysentery (Ur:1), gastric (Ur: 15), intestinal infection (Ur: 4), constipation (Ur: 5), (Ur: 4), tapeworm (Ur: 7), TB (Ur: 3), typhoid (Ur:1), vomiting (Ur: 3), digestive problem (Ur: 3)	60	53	0.12
Genitourinary system diseases including STD: (gonorrhea (Ur: 4), infertility (impotence) (Ur: 7), male sterility (Ur:1), mate organ burn (Ur:1), abortion (Ur: 2), placenta problem (Ur:1), uterine bleeding (Ur:9)	25	21	0.16
Blood and circulatory-related disease: (diabetes (Ur: 9), hepatitis (Ur: 6), bleeding or clotting problem) (Ur:5), blood vessel problems (Ur: 3), cholesterol (ur:2), heartburn pain (Ur: 11), hypertension (Ur:13), kidney stone (Ur: 8), stroke (Ur:1)	58	39	0.33
Nervous system and Cancer-related disease: (bone rupture (Ur:2), breast cancer (Ur: 1), breast pain (Ur: 1), cancer (Ur:12), mastitis (Ur:1), arthritis (Ur:3), back pain (Ur:1), nerve/paralysis) (Ur: 5, depression (Ur: 4), epilepsy (Ur:1), mental illness (Ur:1)	32	28	0.13

As shown in Table 5, some of the specific diseases reported frequently have high-use reports, implying that Gamo herbalists mostly treat them. Among others, skin-related disease is the leading case, having 47 use reports, followed by wound cases, having 29 use reports, headache, 27 use reports, and malaria, having 15 use reports. This might be related to the lifestyle of local communities, which is mainly agricultural farming. Other cases, including breast pain, mastitis, typhoid, bronchitis, insect bites, and epilepsy, have low use reports (1) and are among traditionally treated conditions. The analysis of the results demonstrated the informants' consensus to determine the efficacy of reported species for a disease category, which might pave the way for searching the pharmaceutic potential of these species by identifying active compounds.

The study reported that the plants with the highest number of use reports were *Ocimum lamiifolium* with 56 reports, *Moringa stenopetala* with 51 reports, *Acmella caulirhiza* with 41 reports, and *Croton macrostachyus* with 40 reports. These could be attributed to their ability to adapt to local environments and their effectiveness in conventional uses. Moreover, *Ensete ventricosum, Manihot esculenta* and *Moringa stenopetala* are widely used for food and fodder in the study area. *Coffea arabica* (with 27 use reports) and *Artemisia absinthium* (with 14 use reports) were used for commercial and cultural ceremonies. Some species, such as *Nicotiana tabacum* (with 36 use reports), *Hagenia abyssinica* (with 35 use reports), *Echinops kebericho* (with 27 use reports), and *Echinops amplexicaulis* (with 23 use reports), were used solely for their medicinal values.

The potency of species that have higher usage reports is likely correlated with the presence of secondary active metabolites, which make them effective in inhibiting various oxidations and biological activities. The prevalence of bioactive compounds such as terpenoids, flavonoids, tannins, saponins, steroids, and essential oils containing linalool, 1-octen-3-yl-npropionate, and 3,7,11-trimethyl-(E, E)-2,6,10-dodecatrienal makes *Ocimum lamiifolium* more effective in treating different diseases [40, 41, 69]. *Moringa* species possess functional bioactive compounds, including phenolic acids, flavonoids, alkaloids, phytosterols, minerals, and organic acids, which make them highly effective in inhibiting multiple biological activities. These activities include antiproliferation, hepatoprotective, anti-inflammatory, antinociceptive, antiperoxidative, cardioprotective anticancer, anti-ulcer cardiovascular, anti-obesity, antiepileptic, antiasthmatic, antidiabetic, anti-allergic, anthelmintic, wound healing, antimicrobial, immunomodulatory, and antidiarrheal properties [70,71].

Compounds such as alkaloids, tannins, saponins, terpenoids, and steroids, along with 4-hexen-1-ol, (E), bis(2-ethylhexyl) phthalate, [1,1'-biphenyl]-2-acetic acid, epizonarene, cyclopentene, 3-isopropenyl-5,5-dimethyl, and 3-carene, might contribute to the healing potency of *Croton macrostachyus* [72,73]. A review study on the anti-malarial effects of the species revealed the existence of cyclohexane diepoxides, such as crotepoxide, lupeol, and betulin; cis-clerodane; crotomacrine; 3β-acetoxytetraxer-14-en-28-oic acid; trachylina-19-oic acid; and trachylina-18-oic acid, which contributed to a higher anti-malarial effect [74].

Traditional healers in Gamo use different plant species to cure specific diseases. Informants identified the top ten plant species that have the highest potential to heal, along with their fidelity level (FL) score. The plants with high healing potential include *Ocimum lamiifolium*, which is effective in treating febrile illness, with an FL score of 1. *Withania somnifera* is useful for curing the evil eye, locally called Buda, to mean spiritual problem with a score of FL: 1. *Hagenia abyssinica*, *Tamarindus indica*, and *Acmella caulirhiza* are effective in treating tapeworm, wound healing, and febrile illness, respectively, with a score of 1. *Echinops. kebericho* is helpful for cancer and sudden illness, with a score of FL: 0.93. *Schefflera abyssinica* is effective for wound healing, with a score of FL: 0.91. *Zingiber officinale* is useful for tonsillitis, with an FL score of 0.87. *Jasminum grandiflorum* is effective for earache and cough, with a score of FL: 0.86. *Eucalyptus globulus* is helpful for febrile illness, with a score of FL: 0.86.

The Gamo healers prefer some species over others when preparing remedies for specific ailments. Six key respondents ranked their preference of potent species for curing certain conditions. This preference could help herbalist to select most effective species over others to prepare effective remedies for better treatments. Furthermore, it also shows the potential species for specific ailments. *Jasminum grandiflorum* (36 scores), *Carissa spinarum* (35 scores), and *Croton macrostachyus* (34 scores) were preferred for treating skin diseases over other plant species, including *Withania somnifera* (31 scores), *Commelina benghalensis* (26 scores), *Datura stramonium* (24 scores), *Vernonia amygdalina* (15 scores), and *Paullinia pinnata* (13 scores). While *Acmella caulirhiza* (76 score) and *Coffea arabica* (70 score) are preferred for their wide use for their wound-healing capacity over *Xanthium strumarium* (60 score*)*, *Argemone mexicana* (59 score), and *Leptadenia hastata* (53 score). *Croton macrostachyus* (75 score) is preferred for its effective wound and skin disease healing capacity. The more preferred species have a significant role in treating diseases in the study area, and these might probably open a door for further studies focusing on identifying specific species trial and cytotoxicity tests for their effectiveness against stated ailments.

### Educational level, age, and traditional knowledge

Our research has revealed a strong correlation between age and knowledge of ethnobotany. Individuals aged over 40 were found to have a better understanding (Table 2). In addition, males tend to have higher exposure to ethnobotanical knowledge than females due to local cultural practices. Interestingly, illiterate people tend to rely more on medicinal plants, while those who are educated prefer modern drugs. Gamo healers pass their knowledge to their first son verbally. These oral transfers are likely because respondents cannot document due to illiteracy. It is important to note that younger residents require more knowledge about ethnobotany to preserve this valuable traditional knowledge [57].

### Major threats and conservation of Gamo medicinal plants

Six major factors threatening Gamo medicinal plants were mentioned by respondents as shown in Table 6. Key respondents were coded as R (respondent) to rank the major threatening factors. Those factors ranked based on their devastating impact on medicinal plants and natural resource as whole. Among others, deforestation, agricultural expansion, and drought were ranked main threatening factors to local biodiversity including medicinal plants. Others such as charcoal use and construction ranked low as the study area is a remote rural area. This is another significance of the present study contributes to environmental protection by prioritizing major threatening factors, which might help to halt further degradation of environment and to set appropriate conservation and managements action to protect further species and natural resource loss in the region.

**Table 6:** Major threatening factors of medicinal plants

Factors	Respondents (R)						Total	Rank
	R1	R2	R3	R4	R5	R6		
Deforestation	6	5	6	4	4	6	31	1st
Drought	4	6	5	6	1	4	26	3rd
Construction	3	3	4	3	2	3	18	4th
Charcoaling	2	1	2	1	5	2	13	5th
Agriculture expansion	5	4	3	5	6	5	28	2nd
Over-exploitation	1	2	1	2	3	1	10	6th

Herbalists frequently assert that they used to gather therapeutic plants close to their homes in the current study area. Currently, nevertheless, gathering TMPs is difficult due to the loss of surrounding forests and drought caused by climate change is hurting the local ecology and medicinal plant species. According to several reports from different regions of the nation, these concerning features indicate common issues [30, 33, 51, 58].

## Conclusions and recommendations

The present survey is the first ethnobotanical study to record the medicinal plants of the Gamo people in the Boreda Abaya District of southern Ethiopia. Traditional knowledge of medicinal plants used by Gamo people has been documented for future research and contributes with significant remarks for updating the country's knowledge of medicinal plants. It also highlights the importance of traditional medicine in the primary health care system. Identifying potential therapeutic species might promote protection of local health care and used for further studies. Documentation of this valuable knowledge will aid in preserving traditional medicine practices, conserving threatened species, and contributing to potential drug discovery in the future. In addition, exploring unassessed area aids in enriching flora and cultural uses of medicinal plants, which aids to compile herbal medicine of the region. The diverse array of medicinal plants, along with their traditional applications, provides a valuable foundation for further exploration, conservation, and potential integration into modern healthcare system. This could be possible through identification of essential therapeutic species and investigation of pharmacological and biological activities. This could enhance local and national health system and promote further scientific research. The present study also highlights the role of traditional knowledge in conserving biological resource.

The present study highlighted about188 medicinal plant species used by local communities utilized for human and domestic animal ailments in Boreda Abaya District. Traditional knowledge of using, preparing, and applying remedies for these medicinal plants was documented. The information might be useful as the baseline for future investigation of new medicinal resources. Many Gamos medicinal plants are herbaceous and harvested from the wild. Gamo herbalists frequently utilized the leaves and seeds of different species. Men have a greater role than women in traditional medicine due to cultural perspectives. Higher proportions of residents were farmers and illiterate and had more ethnobotanical knowledge than educated and young people. This might relate to their exposure and experience, and it indicate formal education is scarce. A higher number of taxa (Nt: 61) were utilized to treat the dermal disease category (ICF: 0.2), while fewer taxa (Ur: 21) were used for the genitourinary disease category (ICF: 0.16). Skin-related disease and wounds are two major conditions having higher use reports in the present study area. Among other recorded species, *Ocimum lamiifolium* (56 use reports), *Moringa stenopetala* (51 use reports), *Acmella caulirhiza* (41 use reports), and *Croton macrostachyus* (40 use reports) have higher use reports for healing different diseases, indicating their effective healing potential. It is time to conduct and widen the pharmaco-chemical studies and safety tests of Gamo medicinal plants.

Further isolating and characterizing the chemical compounds and pharmacological tests are necessary for species having higher use reports. Conservation agencies and local governments should focus on traditional medicinal plant conservation and documentation of the people's cultural knowledge of ethnobotany. Providing conservation priority, promoting conservation methods like field gene banks, Arboretums, and Botanical gardens, and supporting traditional healers might help halt the rapidly diminishing medicinal plants. Furthermore, more public awareness is needed to encourage the local people to manage and sustainably utilize medicinal plant resources.

## Data Availability

The authors declare that all other data supporting the findings of this study are available within the article and its supplementary information files.

## Abbreviations

EBI: Ethiopian Biodiversity Institute
SBGH: Shashemene Botanical Garden Herbarium
TMPs: Traditional medicinal plants
TM: Traditional medicine
DA: Development agents
CK: Community knowledge
Ur: Use reports
ICF: Informant consensus factor
NDA: Natural database for Africa
STDs: Sexual transmitted diseases
FL: Fidelity level
